# A high throughput DNA extraction method with high yield and quality

**DOI:** 10.1186/1746-4811-8-26

**Published:** 2012-07-28

**Authors:** Zhanguo Xin, Junping Chen

**Affiliations:** 1 Plant Stress and Germplasm Development Unit, USDA-ARS, 3810 4th Street, Lubbock, TX, 79415, USA

**Keywords:** DNA extraction, CTAB, MagAttract

## Abstract

**Background:**

Preparation of large quantity and high quality genomic DNA from a large number of plant samples is a major bottleneck for most genetic and genomic analyses, such as, genetic mapping, TILLING (Targeting Induced Local Lesion IN Genome), and next-generation sequencing directly from sheared genomic DNA. A variety of DNA preparation methods and commercial kits are available. However, they are either low throughput, low yield, or costly. Here, we describe a method for high throughput genomic DNA isolation from sorghum [*Sorghum bicolor* (L.) Moench] leaves and dry seeds with high yield, high quality, and affordable cost.

**Results:**

We developed a high throughput DNA isolation method by combining a high yield CTAB extraction method with an improved cleanup procedure based on MagAttract kit. The method yielded large quantity and high quality DNA from both lyophilized sorghum leaves and dry seeds. The DNA yield was improved by nearly 30 fold with 4 times less consumption of MagAttract beads. The method can also be used in other plant species, including cotton leaves and pine needles.

**Conclusion:**

A high throughput system for DNA extraction from sorghum leaves and seeds was developed and validated. The main advantages of the method are low cost, high yield, high quality, and high throughput. One person can process two 96-well plates in a working day at a cost of $0.10 per sample of magnetic beads plus other consumables that other methods will also need.

## Introduction

Extraction of large quantity and high quality DNA is often a limiting factor in genetic analysis of plant traits important to agriculture. For example, TILLING (Targeting Induced Local Lesions IN Genomes) has become an increasingly popular reverse genetic tool to identify mutation series for genes with known sequence [1-5]. A major limitation for TILLING is to obtain a large quantity of genomic DNA from thousands of individual lines. The quality of DNA from each line must be consistent from sample to sample to allow equal pooling of DNA from several individuals. Many high throughput methods to isolate DNA from plant tissues are available; however, these methods produce either insufficient amounts or inconsistent quality of DNA for TILLING [6-10]. Several commercial kits are also available to extract genomic DNA from plant tissues with sufficient quality [11], but the yield of DNA produced from commercial kits is often low. Moreover, the cost can be prohibitive for small laboratories.

Here, we present a low-cost, high yield, high quality, and high throughput method to prepare genomic DNA from sorghum [*Sorghum bicolor* (L.) Moench] leaves and dry seeds. The method combines a traditional CTAB DNA extraction and modified DNA clean-up procedure using silica-coated magnetic beads into a new platform that reduced significantly the use of the expensive magnetic beads while producing high yield and high quality of genomic DNA. The applicability of this method is also tested in several other plant species available in the laboratory.

## Materials

Seeds and leaf tissues used for this study were harvested from sorghum plants [*Sorghum bicolor* (L.) Moench cv. BTx623] grown on the experimental field of USDA-ARS in Lubbock, Texas. During growing season approximately 5 to 20 cm^2^ section of sorghum leaf (0.1 to 0.4 g fresh weight) was sampled into a labeled 2 ml microfuge tube. All collected samples were kept on ice in the field. After returning to laboratory, the leaf samples were then placed into a Labconco freeze dryer and lyophilized for two days. The dried leaf samples were either used directly for DNA extraction or stored at room temperature in dark for up to three months. Eight to twelve dry sorghum seeds were directly used for isolation of genomic DNA.

A variety of plant species available on the campus of the Plant Stress and Germplasm Research Unit, USDA-ARS, at Lubbock, Texas were used to test the applicability of this method to other species. Bermuda Grass [*Cynodondactylon* (L.)], poplar [*Populus deltoides*, (L.)], Eastern cottonwood], pine [*Pinus eldarica* (L.), Afghan pine] are lawn grass and ornamental plants on the campus. Newly developed leaves or needles were sampled into 2-ml microfuge tubes and lyophilized. Flag leaves that were still green were taken from field-grown wheat [*Triticum astivum* (L.)], which were near maturity. Young developing leaves were used for maize [*Zea mays* (L.)] seedlings that were planted in the field two weeks ago. Cotton [*Gossypium hirsutum* (L.)] and tobacco [*Nicotiana tabacum* (L.)] were grown in greenhouses. Newly developed sorghum leaves were taken from greenhouse grown sorghum inbred line BTx623 to serve as a control. To determine the yield of genomic DNA, the microfuge tubes were weighed before sampling and after the plant tissue was lyophilized. Dry weight of plant tissues was determined by subtracting the tube weight from the final weight. Genomic DNA yield was expressed as μg DNA per mg of dried tissue. Weighing plant tissue is not necessary for routine processing of samples.

### Reagents and consumables

· CTAB Hexadecetyltrimethylamonium Bromide (Sigma, Cat# H6269).

· MagAttract Plant DNA core kit (Qiagen, Cat# 67163).

· MagAttract suspension G (Qiagen, Cat# 1026901).

· Ethanol.

· RNase A (Sigma, Cat# R4875).

· 5 mm Tungsten beads (www.maximum-velocity.com/index.htm, Cat # 5040).

· Safe-Lock microfuge tubes 2.0 ml (Eppendorf, Cat # 022363352).

· Flat bottom 96-well microtiter plates (Greiner, Cat # 655101).

· Microtiter plate sealing mat (VWR, Cat # 82006–692).

· PCR plates (Labsource, Cat# T53-401).

### Solutions

· DNA extraction buffer: 100 mM Tris (pH 8.0), 20 mM EDTA, 2% CTAB, 1.2 M NaCl, and 0.1% β-mercaptoethanol (add before use). After adding β-mercaptoethanol, the extraction buffer must be used within a day. β-mercaptoethanol is toxic and has strong smell must be disposed to special container under Laminar flow hood.

· Dilution buffer: 100 mM Tris (pH8.0), 20 mM EDTA, 2% CTAB.

· High salt TE: 10 mM Tris (pH8.0), 2 mM EDTA, 1 M NaCl (add RNase A at 50 μg ml-1).

· Chloroform:isoamylalcohol at 24:1 v/v. Chloroform is corrosive and toxic and must be handled under Laminar flow hood.

· Wash buffer: Add 300 ml TE to 700 ml ethanol.

· TE: 10 mM Tris, 1 mM EDTA, pH 8.0.

### Equipment

· Labconco freeze dryer (Millrock Technology Inc. Kingston, New York, USA).

· TissueLyser II (Qiagen, Cat # 85300).

· Multichannel pipette.

· Oven or heating block, any type that can maintain temperature at 60°C.

· Sorvall Legend RT plate centrifuge (Harlow Scientific, Arlington, Massachusetts, USA) or any lab top centrifuge.

· 96-well magnet type B (Qiagen Cat # 9012916).

· Nanodrop ND-1000 Spectrophotometer (NanoDrop Technology, Wilmington, Delaware, USA).

· TECAN infinite M200 microplate reader (Tecan US, Inc.Durham, North Carolina, USA).

### Protocol

1. Add one tungsten ball to the lyophilized leaf tissue (~10 to 40 mg) or dry seeds (10 seeds ~300 mg) in 2-ml microfuge tube. Place tubes in the grinding racks. Each rack holds 24 tubes. The two racks must hold equal number of tubes for balance.

2. Grind tissues at 28 strokes per second for 1 minute using TissueLyser II, repeat 3 more times, switching orientation each time for even grinding.

3. Add 750 μl extraction buffer to the ground tissue using multichannel pipette.

4. Cap the tubes and mix twice for 30 s at 20 strokes per second and incubate at 60°C for 1 hour.

5. Cool down tubes at room temperature for 5 min, then add 750 μl Chloroform:isoamylalcohol (24:1, v/v) to each tube, mix well, and centrifuge at 3000 g for 15 minutes.

6. Transfer aqueous layer (~500 μl) to a new set of labeled tubes.

7. Add 1 ml dilution buffer to the aqueous phase.

8. Mix well and incubate at 60°C for 30 minutes (Note: copious amount of precipitate of DNA-CTAB complex should be observed at the end of the incubation).

9. Centrifuge at 3000 g for 15 minutes and discard the supernatant.

10. Add 1 ml washing buffer to the pellet and soak it at RT for 30 minutes to remove access CTAB.

11. Centrifuge at 3000 g for 15 minutes and discard the supernatant.

12. Re-suspend DNA pellet in 100 μl high salt TE with RNase A and incubate at 60°C for 30 minutes.

13. Transfer the high salt TE DNA solution to 96-well micortiter plate, one sample per well.

14. Add 5 μl MagAttract suspension G solution to each well using a multichannel pipette.

15. Add 120 μl 100% ethanol to each well.

16. Cover the microtiter plate tightly with a silicone plate sealing mat and mix gently. Incubate it at room temperature for 5 minutes (to allow DNA adhere onto the surface of beads).

17. Place the DNA plate on Magnet B to hold the MagAttract beads and then pour off the ethanol solution.

18. Wash the beads three times with 200 μl washing buffer and air-dry the beads for 10 minutes at RT.

19. Add 100 μl TE to each sample well to re-suspend DNA.

20. Incubate at 60°C for 5 min and mix gently (to allow the bound DNA release into TE solution).

21. Place the plate on Magnet B and transfer DNA solution to a new 96-well plate.

22. Quantify DNA on a Nanodrop spectrophotometer or TECAN plate reader.

## Result

**Figure 1:**
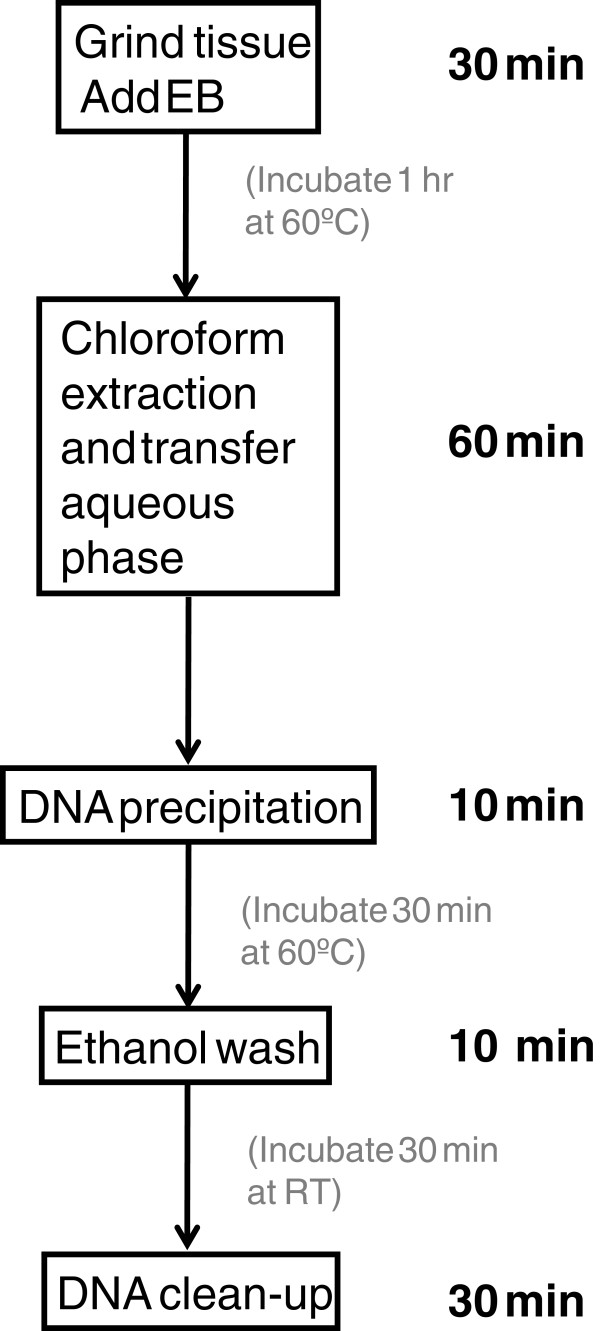
**Flowchart of five major steps of the DNA extraction method.** It takes 2 h and 20 minutes hand-on time to complete a 96-well plate. One technician can process two 96-well plates in a working day.

The procedure to extract genomic DNA is illustrated in a flowchart in Figure 1. The method has five major steps. 1. Grinding plant tissue. We routinely grind lyophilized tissue in Tissuelyser, which can process 48 samples a time. Fresh tissue can also be ground with mortar and pestle but the throughput is much lower. 2. Chloroform extraction. This is a critical step to increase the recovery of aqueous phase and DNA yield. 3. Precipitation of DNA with CTAB dilution buffer. At this step, large amount of DNA-CTAB complex should be observed. It is a key step to know if good DNA yield will be achieved. 4. Wash DNA-CTAB complex with ethanol wash. This step is to wash out the excess CTAB from the DNA-CTAB complex. This is a critical step for efficient binding of DNA to MagAttract beads. 5. DNA cleanup. The hand-on time for preparing one 96-well plate DNA samples is less than 2.5 hours. One technician can easily process two 96-well plates of DNA samples in a working day.

Results in Table 1 showed the differences in DNA yield and DNA quality between the commercial MagAttract kit and the method described in this study. With this improved extraction method, DNA yield was increased by an average of 30 folds with consistently high purity (Table 1). The kit produced at best 3 μg total DNA per leaf sample, and the purity of DNA was poor and inconsistent as indicated by the variable ratios of OD_260νμ_/OD_230νμ_. No detectable DNA was isolated from dry seeds using the kit (Table 1).

**Table 1:** Comparison of DNA yield and quality between the MagAttract kit and this improved method

**Tissue Type**	**Method**	**Total DNA Yield (μg per sample)**	**OD**_**260nm**_**/OD**_**280nm**_	**OD**_**260nm**_**/OD**_**230nm**_
Leaves	MagAttract kit	1.7 ± 0.4^†^	2.16 ± 0.22	0.65 ± 0.09
	This method	49.8 ± 9.1*	1.88 ± 0.02	2.35 ± 0.03
Seeds	MagAttract kit	N/A	N/A	N/A
	This method	30.5 ± 12.1*	1.90 ± 0.02*	2.24 ± 0.11*

† Average yield of 14 successful extractions and standard deviation.

* Average yield of a 96-well plate and the standard deviation.

**Figure 2:**
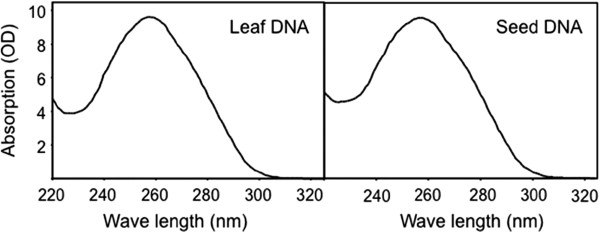
**Absorption Spectrum of DNA isolated from lyophilized sorghum leaves and dry seeds.** DNA isolated from leaves or seeds was diluted to 500 ng/μl with TE and scanned on NanoDrop from 220 to 400 nm.

**Figure 3:**
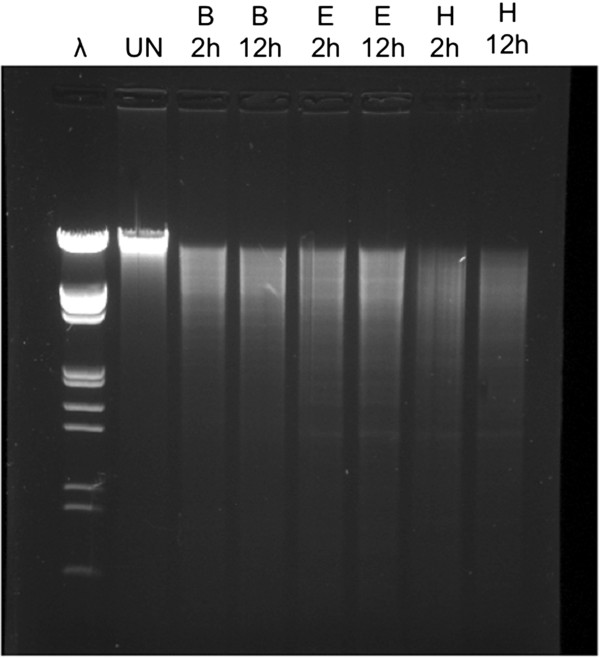
**Restriction digested and undigested sorghum leaf DNA.** Five μg leaf DNA was digested in 20 μl reaction volume for 2 or 12 hours with 5 units of Bam HI (B), Eco RI (E), or Hind III (H). About 5 μl digestion was loaded each lane. UN, undigested leaf DNA, λ, phage λ DNA marker cut with Eco RI and Hind III. Similar digestion kinetics was observed in DNA extracted from dry seeds.

To examine the quality of DNA, we scanned the DNA samples from 220 to 400 nm on a Nanodrop NP-1000. The absorption spectrum of the DNA extracted from both leaf and seed tissues closely resembled the spectrum of pure λ phage DNA (Figure 2). The quality of the DNA was further tested by electrophoresis of the DNA on a 1% Agarose gel before and after restriction digestion with Bam HI, Eco RI, and Hind III. As shown in Figure 3, the genomic DNA fragment isolated with our method was over 20 kb and could be cut to completion by common restriction enzymes within 2 hours. The results suggest that our improved DNA extraction method produces high quantity and high quality genomic DNA from sorghum leaves and dry seeds.

**Figure 4:**
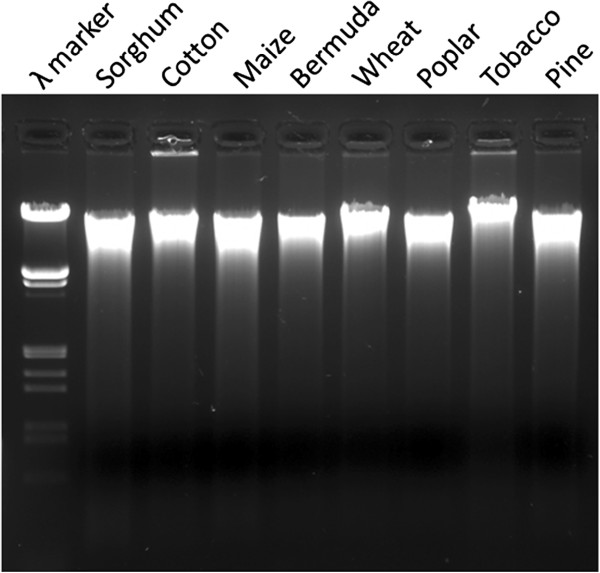
**Genomic DNA isolated from eight plant species.** Two μg of genomic DNA isolated from each of the seven plants species was separated on 1% Agarose gel in 0.5X TBE and 0.5 μg^.^ml^-1^ ethidium bromide.

To test if the method we developed using sorghum leaves and seeds is applicable to other plant species, we collected leaf samples from all plant species that were accessible at the moment. They included Bermuda lawn grass, tobacco, maize and wheat, two important grain crops, poplar, a promising bioenergy plant, cotton and pine needles, two species that are considered difficult to isolate high quality genomic DNA due to the presence of polysaccharides and phenolic compounds [12-14]. The genomic DNA yield and quality were showed in Table 2. DNA yield varied among species. Greenhouse-grown tobacco leaves produced an average yield of 4.7 μg per mg dry leaf material, probably due to its large genome size and young and tender tissue [15]. Poplar leaves produced the lowest yield at 0.5 μg per mg dry leaf tissue, most likely due to its small genome size and hardy tissue [16]. Despite of the large variation in DNA yield, the DNA quality, as evidenced by the ratios of OD_260nm_ to OD_280nm_ and to OD_230nm_ and the gel image (Figure 4), were largely consistent. The success of this method in obtaining high quality genomic DNA from all eight plant species tested demonstrated the broad applicability of this method.

**Table 2:** Yield and quality of genomic DNA isolated from a variety of plant species

**Plant Tissue**	**DNA yield (μg/mg dry tissue)**	**OD**_**260nm**_**/OD**_**280nm**_	**OD**_**260nm**_**/OD**_**230nm**_
Sorghum leaves	1.5±0.43	1.85±0.02	2.46±0.13
Cotton leaves	1.0±0.11	1.85±0.01	2.25±0.08
Maize leaves	2.6±0.20	1.81±0.01	2.48±0.04
Bermudagrass leaves	1.0±0.15	1.82±0.02	2.49±0.03
Wheat leaves	2.5±0.42	1.85±0.02	2.46±0.04
Poplar leaves	0.5±0.06	1.80±0.01	2.46±0.09
Tobacco leaves	4.7±0.82	1.87±0.01	2.62±0.03
Pine needles	1.1±0.19	1.78±0.06	2.71±0.40

The values in the table are average of four samples plus and minus standard deviation.

## Discussion

We presented a high throughput, high-yield, and cost-effective method to extract high quality genomic DNA from a variety of plant species. This method works in single tube format, as well as, in 96-well plate format. We routinely grind tissue in 48 individual 2 ml microfuge tubes and conduct DNA cleanup in 96-well plate. For a small number of samples, all procedures can be performed with individual tubes using a regular magnet to attract the MagAttract beads to the tube wall during DNA cleanup. For large number of samples, tissue can also be ground in 96-well plate format with substantial increase in throughput. However, extreme caution must be exercised to avoid cross contamination of ground samples among adjacent wells.

The original MagAttract kit calls for the use of fresh plant tissue. The fresh tissue is ground in liquid nitrogen-cooled plates. The ground tissue is immediately mixed with 300 μl proprietary lysis buffer RLT. After centrifugation, 200 μl supernatant is transferred to a 96-well plate containing 65 μl binding buffer RB and 20 μl MagAttract beads each well for DNA cleanup. The use of fresh tissue and liquid nitrogen cooled plates reduce the throughput. The claimed DNA yield from the instruction manual is 1 to 15 μg DNA per sample. In our hand, most samples yielded less than 2 μg per leaf sample; the best result achieved was 3 μg per sample. We did not obtain detectable amount of DNA from dry seed samples. Our method used lyophilized plant tissues, which allowed the samples ground under room temperature, significantly increased the throughput, DNA yield, and DNA quality.

We made three critical modifications to the kit. First, a chloroform extraction step was added after CTAB extraction buffer (similar to lysis buffer). This one-step chloroform extraction significantly improved the DNA yield and quality. For example, without this step, we were barely able to recover 200 μl of the aqueous phase out of 750 μl extraction buffer added to lyophilized tissue. By adding a chloroform extraction step, we could easily recover over 600 μl of the aqueous phase. In high throughput format, we routinely took 500 μl of the aqueous phase from each sample with an 8-channel pipette without the risk of contamination from the organic phase. In addition, chloroform extraction removes polysaccharides, lipids, and other nonpolar substances from aqueous phase, resulting in cleaner DNA [17]. The second improvement was the increased efficiency of CTAB-DNA precipitation and pellet re-suspension by bringing down the NaCl concentration in extraction buffer with CTAB dilution buffer. Most CTAB DNA extraction methods reported in literature [9,10,13,18] are variations of the method reported by Doyle and Doyle [19], in which DNA in CTAB extraction buffer is precipitated by adding 0.5 volume isopropanol. In our experience, these methods vary in DNA precipitation efficiency among samples and produce inconsistent DNA pellets. Sometimes, no DNA pellet was formed with certain type/amount of samples, such as seed samples in our experiment. In addition, it is usually very difficult to re-dissolve the pellet. We found that the method originally described by Dutta et. al. in 1953 [20] worked the best. To precipitate the CTAB-DNA complex, the aqueous phase DNA extracts was diluted with 2 volumes of dilution buffer, bringing the NaCl concentration from 1.2 M (extraction buffer) down to about 0.4 M. This modification had consistently allowed the precipitation of copious amount of CTAB-DNA complex in a range of plant samples that vary in tissue types, sample sizes, and plant species. The solubility of the CTAB-DNA pellet was improved drastically as the results of the first two modifications. The third improvement made was the efficient DNA cleanup procedure in presence of 1 M NaCl and 50% ethanol without using hazardous chaotropic salt, such as guanidine chloride. Under this condition, DNA can bind efficiently to the beads and released completely in TE. In addition, genomic DNA recovered from this cleaning process had little contamination by polysaccharides and proteins as indicated by the ratios of OD_260_/OD_230_ (>2) and OD_260_/OD_280_ (between 1.8 and 2.0). Moreover, with the modified binding condition, the amount of MagAttract bead suspension needed for each DNA sample was reduced by 4 folds, from 20 μl (as required by the kit) down to only 5 μl. Thus, the cost for preparing DNA is dramatically reduced. Now, the MagAttract beads (MagAttract suspension G) can be purchased separately from the kit. With this modified method, the cost of MagAttract beads was reduced to less than $0.10 per sample.

With these modifications made in DNA extraction and cleanup procedures, we were able to isolate large amounts of DNA with consistent high quality. In a 96-well plate format, every sample on the plate produced sufficient DNA for our TILLING experiments (Table 1). In general, this method yielded consistently 30 times more DNA than the kit tested. In addition, this modified method worked in a wide range of sample sizes, while most commercial kits have tight constriction on sample size. As indicated in Table 1, where leaf samples were harvested from field-grown sorghum without weighing and seed samples were taken by volume (8 to 12 seeds). Although the total DNA yield per sample and sample size varied among seed and leaf samples, the DNA quality was consistent.

We tested the suitability of isolating genomic DNA to seven other species, including Bermuda lawn grass, tobacco, maize, wheat, poplar, cotton, and pine. In all species tested, large amount and high quality genomic DNA was obtained. Poplar leaves had the lowest yield (Table 2). However, over 100 mg of dried leaf tissue can be used without compromising DNA quality. With large sample size (>50 mg), over 20 μg genomic DNA could be extracted per sample. Only pine needles presented some difficulty at large sample sizes. The maximum amount of dried pine needles could be used by this method was 30 mg, beyond which DNA yield and quality were severely compromised (data not shown). With 20 to 30 mg dried young needles, we could easily obtain over 20 μg high quality genomic DNA, which is sufficient for most genomic studies. For all other species, including cotton, we did not find detectable decrease in DNA quality for tissue amount up to 100 mg dry weight. In general, the low cost and high throughput method described in this study is suitable for preparation of sufficient high quality DNA for TILLING from plant samples routinely taken from a convenient position without weighing.

## Conclusion

An efficient DNA extraction method for sorghum leaf and seed tissues is described. The method consistently produces high yield and high quality genomic DNA at an affordable cost. This method can be used to extract high quality genomic DNA from a variety of plant species, including cotton leaves and pine needles. The low cost, high throughput, high quality, high yield, and broad applicability of the method make it a useful method for genomic studies that need large quantity and high quality DNA. Especially, the high DNA yield is particularly useful for reverse genetic studies that required to use the DNA to analyze many targets.

## Competing interests

The authors declare that they have no competing interests.

## Authors’ contributions

ZX and JC designed the method, carried out the laboratory work to fine-tune the system. Both authors participated in writing and revising, and approved the final manuscript.

## Disclaimer

Mention of trade names or commercial products in this article is solely for the purpose of providing specific information and does not imply recommendation or endorsement by the U.S. Department of Agriculture. USDA is an equal opportunity provider and employer.
